# Root and microbial contributions to anoxic microsite formation in the rhizosphere: a microfluidic approach

**DOI:** 10.1111/nph.71109

**Published:** 2026-03-20

**Authors:** Emily M. Lacroix, Giulia Ceriotti, Daniel Garrido‐Sanz, Sergey M. Borisov, Jasmine S. Berg, Christoph Keel, Pietro de Anna, Marco Keiluweit

**Affiliations:** ^1^ Institute of Earth Surface Dynamics University of Lausanne Lausanne 1015 Switzerland; ^2^ Department of Earth Sciences Dartmouth College Hanover 03755 NH USA; ^3^ Department of Fundamental Microbiology University of Lausanne Lausanne 1015 Switzerland; ^4^ Department of Biology Autonomous University of Madrid Madrid 28049 Spain; ^5^ Institute of Analytical Chemistry and Food Chemistry Graz University of Technology Graz 8010 Austria; ^6^ Institute of Earth Sciences University of Lausanne Lausanne 1015 Switzerland

**Keywords:** anaerobic, anoxic microsites, microfluidics, oxygen, rhizosphere, root‐on‐a‐chip, suboxia

## Abstract

- Plant root‐associated anoxic microsites may influence the fate of nutrients and contaminants in the rhizosphere, but their dynamics remain relatively unknown.
- To examine the formation of root‐induced anoxic microsites over space and time, we use microfluidic devices integrated with transparent, planar oxygen sensors in a wheat (*Triticum aestivum*) rhizosphere, with and without soil microorganisms.
- We found that suboxic (< 2% air saturation) conditions commonly establish at root tips and more rarely establish along more mature root segments, particularly in the presence of soil organic matter and complex microbial communities. Additionally, the distribution of oxygen, and thus root‐induced anoxic microsites, depends on complex interactions among light–dark cycles, growth rate, and presence of microorganisms in the rhizosphere.
- This study provides real‐time observations of the micron‐scale oxygen dynamics around actively growing roots, thereby linking root physiology to anoxic microsite formation in the rhizosphere. Our work suggests a strong potential for root‐driven anoxic microsite formation, prompting important questions about anoxic microsite impact on biogeochemical processes in natural rhizosphere soil.

## Introduction

Anoxic microsites are zones of oxygen (O_2_) depletion severe enough to host anaerobic processes in bulk oxic soils and sediments (Lacroix *et al*., 2023). Relatively understudied, anoxic microsites are of enormous relevance to plant nutrient and contaminant acquisition as well as soil carbon cycling. For example, anoxic microsites may host denitrification, which promotes the loss of aqueous nitrate from soils (Sexstone *et al*., 1985; Lacroix *et al*., 2023; Schlüter *et al*., 2025), thereby decreasing plant access to nitrogen. Similarly, anoxic microsites can promote Fe‐oxide dissolution, which can solubilize adsorbed phosphorus, contaminants, and carbon. This Fe‐oxide dissolution can increase plant access to phosphorus and contaminants (Liptzin & Silver, 2009; Malakar *et al*., 2020) and microbial access to previously inaccessible soil carbon (Huang *et al*., 2020; Bölscher *et al*., 2025). Although anoxic microsites are theorized to preferentially establish in the rhizosphere (Lecomte *et al*., 2018), this phenomenon has yet to be broadly demonstrated, and it remains unclear if, when, where, and how anoxic microsites develop near plant roots. As a result, the biogeochemical influence of anoxic microsites near plant roots remains unquantified, and our understanding of plant nutrient and contaminant acquisition and the fate of rhizosphere carbon remains incomplete.

Anoxic microsites form when soil O_2_ demand, largely driven by biological O_2_ demand, is greater than the local supply of O_2_, which is largely dictated by soil moisture and texture (Keiluweit *et al*., 2016). The rhizosphere represents a complex environment of limited O_2_ supply and enhanced O_2_ demand and, thus, a theoretical hotspot for anoxic microsite formation. Although root pore channels can effectively transport O_2_, compressed soil particles surrounding root pore channels may experience limited O_2_ supply (Carminati *et al*., 2009; Aravena *et al*., 2011; Phalempin *et al*., 2021). Moreover, high concentrations of mucilage, a gel‐like root exudate, within the rhizosphere can enhance localized water retention, further limiting O_2_ diffusion (Ahmed *et al*., 2014). In terms of O_2_ demand, microbes utilize O_2_ to respire soluble organic compounds exuded from root tips and emerging lateral roots during periods of photosynthetic activity (Cardon & Gage, 2006; Sasse *et al*., 2020; Tsai *et al*., 2025). At the same time, roots consume O_2_ to support cellular root respiration (Højberg & Sorensen, 1993). However, the O_2_ demand of roots vs rhizosphere microorganisms and this ratio's dependence on root morphology remain relatively unknown.

In addition to theory, empirical evidence suggests that the upland rhizosphere harbors anoxic microsites. Whereas O_2_ concentrations near wetland plant roots are elevated compared to the bulk soil (due to radial O_2_ loss from aerenchyma, Larsen *et al*., 2015; Koop‐Jakobsen *et al*., 2017), O_2_ concentrations and/or redox potentials near unsaturated plant roots tend to be lower than nearby bulk soils (Fischer *et al*., 1989; Rudolph *et al*., 2012; Tschiersch *et al*., 2012; Uteau *et al*., 2015; Colmer *et al*., 2020; Bereswill *et al*., 2023). Evidence of anaerobic processes in the rhizosphere, such as denitrification, iron oxide reduction, and methanogenesis, indirectly suggests the presence of root‐associated anoxic microsites in upland soils (Fimmen *et al*., 2008; Schulz *et al*., 2016; Guyonnet *et al*., 2017; Praeg *et al*., 2020; Veelen *et al*., 2020).

Despite this evidence, rhizosphere‐associated anoxic microsites have yet to be incorporated into conceptualizations of rhizosphere biogeochemistry, in part because studying the rhizosphere and anoxic microsites is technically challenging (Ahkami *et al*., 2023; Lacroix *et al*., 2023). Tracking the extent of the rhizosphere is difficult because soils are opaque and roots are constantly growing. The use of rhizoboxes, O_2_ microsensors, microbial O_2_ biosensors, and commercially available O_2_ planar optode systems has advanced our understanding of rhizosphere O_2_ dynamics (Højberg *et al*., 1999; Lenzewski *et al*., 2018; Zimmermann *et al*., 2022; Garcia Arredondo *et al*., 2024). However, O_2_ microsensors only allow for precise measurements in one dimension; microbial O_2_ biosensors can be difficult to produce and image, and commercially available planar optode systems (such as those from PreSens) are opaque, making it challenging to precisely co‐locate roots and rhizosphere soil. Microfluidic approaches to studying rhizosphere dynamics allow for controlled environmental conditions, clear identification of root location, and high‐resolution microscopic imaging of roots and their surroundings (Grossmann *et al*., 2011; Aufrecht *et al*., 2022). Separately, recently developed transparent O_2_ planar optodes integrated with microfluidic devices allow for the co‐visualization of device contents (e.g. microorganisms) and O_2_ (Ceriotti *et al*., 2022). Integrating these approaches to study rhizosphere O_2_ dynamics promises to advance our understanding of root‐associated anoxic microsites.

In this study, we sought to: (1) determine whether roots were key drivers of anoxic microsites; (2) explore spatiotemporal patterns of O_2_ concentration around a growing plant root; and (3) define the relative contribution of microbial vs plant O_2_ demand in driving anoxic microsite formation in the rhizosphere. To achieve these objectives, we performed two experiments, an O_2_‐gradient characterization experiment and a timelapse experiment, examining young wheat roots in microfluidic devices integrated with transparent planar O_2_ sensors and various microbial inocula. We used two O_2_ concentration thresholds to characterize anoxic microsites in our system: hypoxic conditions (1.92 mg l^−1^ O_2_, *c*. 20% air saturation), the threshold below which animals may struggle to survive, and suboxic conditions (0.16 mg l^−1^ O_2_, < 2% air saturation), the threshold below which anaerobic denitrification is expected to occur (Berg *et al*., 2022).

## Materials and Methods

### Device design, construction, and filling

**Fig. 1:**
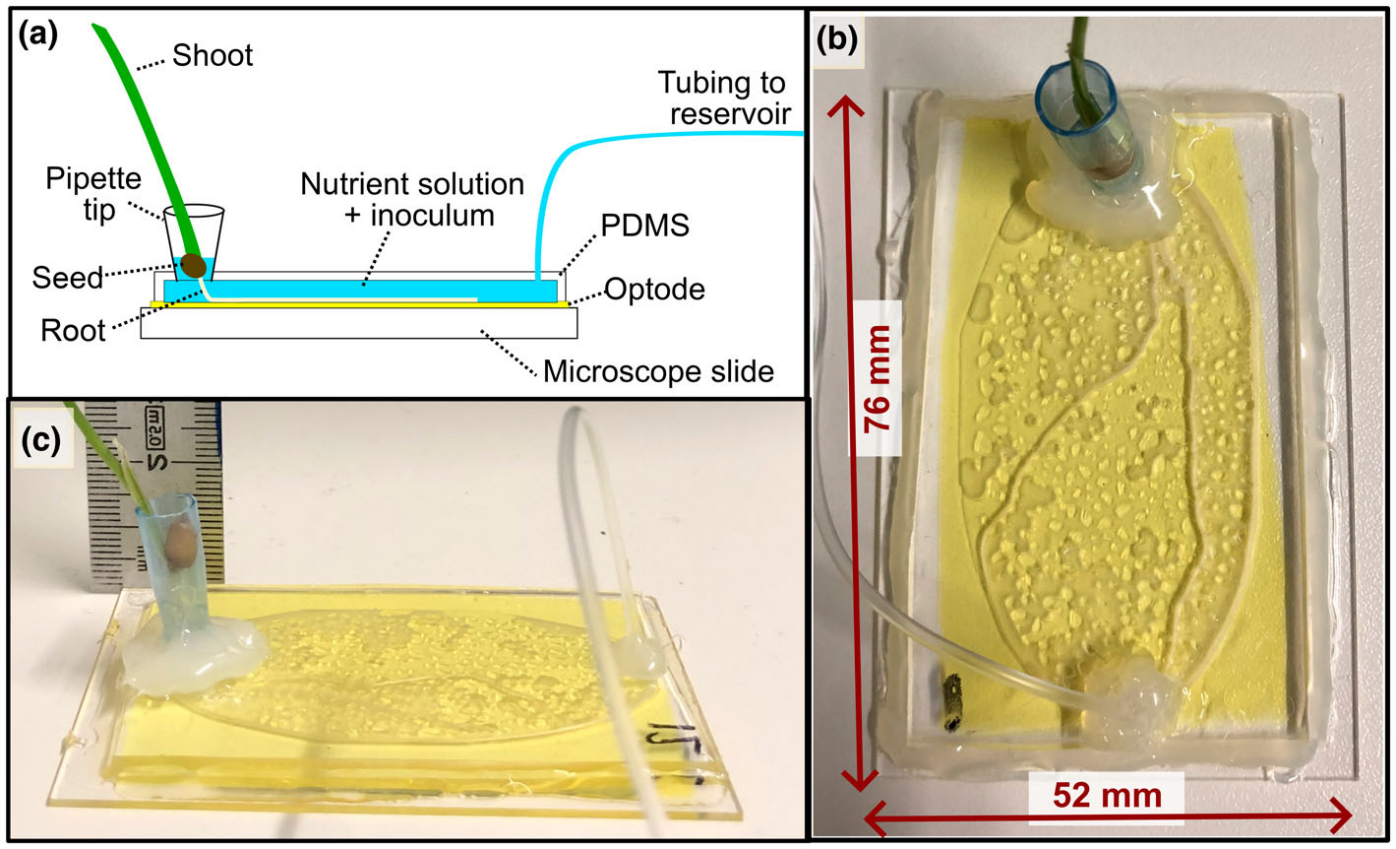
Diagram and photos of microfluidic device. All panels show optode foil in yellow. (a) Side‐view diagram of microfluidic device; (b) photo of microfluidic device, plan view; (c) photo of microfluidic device, side view.

For both experiments, growth devices were constructed using a 76 mm × 52 mm glass microscope slide, overlain with a thin (*c*. 5 μm) layer of transparent, luminescence‐based O_2_‐sensing optode (as described in Ceriotti *et al*., 2022) and topped with a polydimethylsiloxane (PDMS) polymer structure meant to mimic a porous medium (Fig. 1).

The luminescence‐based O_2_‐sensing optodes allow for the creation of microscale oxygen concentration maps from the ratio of the phosphorescence signal from the indicator dye and fluorescence signal from the reference dye (hereafter ‘luminescence ratio’), with the relationship between luminescence ratio and O_2_ concentration following an exponential decay function:

(Eqn 1) $$\left[O_{2}\right]=A\times e^{-2R}+C$$

where R is the luminescence ratio and A and C are constants derived during the calibration procedure (Ceriotti *et al*., 2022).

We used a two‐point calibration scheme for each experimental setup. In brief, glass slide‐mounted, O_2_ optodes from the same batch were affixed with a straight‐channel PDMS piece. The channels were filled with air‐saturated water (9.2 mg l^−1^ O_2_) or chemically anoxic water (0.1 M sodium sulfite with cobalt catalyst, 0 mg l^−1^ O_2_). Within each channel, we obtained 10 images of the reference and sensor dyes using the same image collection conditions utilized in the experiments. For each image, the luminescence ratio of each pixel was calculated, and the ratio was then averaged across the entire image. We fitted Eqn 1 to the luminescence ratios and their associated O_2_ concentrations to determine equation constants A and C. Eqn 1 with calibration‐derived constants was subsequently used to convert luminescence ratios to O_2_ concentrations for all images collected during experiments.

The 2D geometry of the PDMS porous medium was adapted from the work of Aufrecht *et al*. (2022) and replicates the characteristic shape distribution of sand particles found in Kahala, HI (USA). This geometry, previously validated for microfluidics fabrication, was exported from the source as a TIFF file and subsequently converted to an STL file using a free online tool (ImageToSTL). The 2D geometry was then extruded into a 3D structure with a final thickness of 0.5 mm. This design was 3D printed using a Form 3L resin printer (Formlabs) and employed as a mold for microfluidic device fabrication. PDMS components were created by mixing Sylgard 184 Silicone Elastomer mixed with 10 w/w% of a curing agent (supplier: Dow Corning, Midland, MI, USA) and curing at 60°C for at least 4 h. PDMS pieces were trimmed and outfitted with holes for Tygon tubing and modified pipette tips.

Finally, PDMS pieces were plasma‐bound to the microscope slides with O_2_‐optode coatings. Modified pipette tips for seedling support were glued into the appropriate hole, and Tygon tubing was installed to allow for device filling and inoculation.

Microfluidic devices were placed under vacuum for 10–20 min to remove gas from the porous PDMS chip. Devices were then transferred to a sterile biohood where they were saturated with one of three inocula using a sterile siphon comprised of microfluidics tubing, a syringe, and a blunt needle. Devices were filled with 1 ml of inoculum suspension, followed by sterile plant nutrient solution (Hoagland's No. 2 Basal Salt Mixture; Sigma‐Aldrich) until several mm of the pipette tip was submerged. Air bubble entrapment was infrequently observed during device saturation.

Pre‐germinated wheat seeds (see the Seed preparation and germination section) were placed in the wetted pipette tips, and the entire device was placed at a *c*. 30° angle in a high‐humidity chamber. Roots were allowed to grow for an additional 2–4 d to allow for sufficient root establishment into the microfluidic device and onto the O_2_‐sensing foil. During and after root establishment, plants were grown at ambient temperature (18–20°C) in a growth area equipped with a Juwel NovoLux LED 60 White light (6500K, 624 Lumen). Plants received 12 h of continuous or semi‐continuous light (see the Experiment 1: Oxygen gradient characterization and Experiment 2: Root tip timelapse sections) per day. The hydraulic connection between the device and reservoir was maintained after initial filling by pausing siphon flow via a pinch clamp. Plant nutrient solution was replenished as needed by resuming reservoir flow until the seed was rewet. Occasionally, root moisture demand resulted in additional bubble formation.

### Preparing inocula for growth

Microfluidic devices were filled with one of three inocula, hereafter referred to as ‘treatments’: sterile plant nutrient solution (‘Sterile’), pure culture of *Pseudomonas protegens* (strain CHA0), or bulk soil inoculum (‘Soil Community’). *Pseudomonas protegens* CHA0 is a well‐characterized plant‐beneficial bacterium that readily associates with wheat roots (Garrido‐Sanz *et al*., 2023). Together, the three treatments represent a gradient of increased microbial community complexity: microbial absence, a single strain of bacteria, and a complex soil community.

The sterile plant nutrient solution was prepared by mixing Hoagland's No. 2 Basal Salt Mixture (Sigma‐Aldrich) with milliQ water according to manufacturer's protocol and autoclaving the resulting mixture.

The *P. protegens* cell suspension was prepared by culturing *P. protegens* wild‐type strain CHA0 from frozen glycerol stock (100 μl) in growth media (*c*. 5 ml) overnight at 25°C on a rotary shaker (180 rpm). The growth medium was composed of an autoclaved aqueous solution of 25 g l^−1^ Nutrient Broth No. 1 (OXOID) and 5 g l^−1^ Yeast Extract. The resulting bacterial culture was subdivided into four Eppendorf tubes, *c*. 1 ml volume each. Cultures were spun down at 200 ***g*** for 8 min, and the resulting supernatant was removed with a pipette. Pelleted cells were washed three times by adding 1 ml of sterile nutrient solution to each tube, pipetting to mix, and repeating the centrifugation step. After three washes, the optical density of the bacterial suspension at 600 nm (OD_600_) was recorded and washed cells were diluted with sterile nutrient solution to a final volume of 1.0 ml and an OD_600_ = 0.05 (*c*. 10^7^ CFUs ml^−1^). Adequate colonization of the wheat root tips was confirmed in pre‐experiments using the same inoculation conditions, GFP‐labeled organisms, and microscopy (data not shown).

The Soil Community inoculum was prepared by mixing 25 g of fresh, refrigerated soil and 25 ml of sterile nutrient solution in duplicate. Soil properties and sampling have been described previously (Harmsen *et al*., 2024; Garrido‐Sanz & Keel, 2025). Soil and solution were shaken for 1 h in an end‐over‐end shaker. Soil particles were then pelleted by centrifugation at 500 ***g*** for 10 min. The resulting supernatants containing detached microbial cells were pooled and represent a standardized species‐rich natural soil microbial community, as previously characterized (Garrido‐Sanz & Keel, 2025). Presence of microorganisms in the supernatant was confirmed by microscopy. Notably, the final Soil Community inoculum contained unpelleted soil particles and any water‐soluble soil moieties, including *c*. 20 mg l^−1^ of non‐purgeable organic carbon (as measured by TOC‐L; Shimadzu Corp., Kyoto, Japan).

### Seed preparation and germination

We used winter wheat (*Triticum aestivum* L.) seeds sourced from Swiss seed distributor Sativa. Seeds were surface sterilized by exposing them to 70% ethanol for 1 min and then 1% sodium hypochlorite solution for 10 min. Seeds were rinsed three times with sterilized milliQ water and drained. Sterilized seeds were then transferred to a sterile pxetri dish, lined with laboratory paper moistened with sterile milliQ water. Seeds were allowed to germinate for 3–4 d, until the root emerged *c*. 1–5 mm from the seed coat.

### Experiment 1: Oxygen gradient characterization

The first experiment was designed to broadly characterize O_2_ in the synthetic rhizospheres. Plants grown for O_2_‐gradient characterization were kept *c*. 20 cm from the grow lamp under diurnal cycles of 12 h : 12 h, light : dark period. All root portions of the plants were shielded from light using aluminum foil. Devices were brought to the microscope stage daily for imaging, with every device and root imaged after *c*. 6 h of light.

Measurements were performed on an inverted Nikon Eclipse Ti2 (Nikon Corp., Tokyo, Japan), equipped with a 4× and 10× objective and a Hamamatsu Orca‐Flash 4.0 camera (Hamamatsu Photonics, Hamamatsu, Japan). We captured daily images of each plant root for at least 7 d after root emergence (*c*. 12–15 d after initial seed wetting) into the device. Each day, we collected large images of the entire device (4× magnification, tiling individual pictures) as well as each root tip, lateral root, and several other notable features (10× magnification, tiling individual pictures). In total, we collected daily images of 36 roots grown in 10 different devices under three growth conditions. Each image was captured in three optical configurations: one adjusted phase contrast configuration to monitor root morphological features and two fluorescence‐based configurations that captured the response and control signals of the O_2_‐optode. For the fluorescence‐based configurations, excitation light was 440 nm and emission light was filtered using Semrock bandpass emission filters (500 ± 20 nm for the reference and 650 ± 13 nm for the O_2_‐sensitive signals). The software used to capture and export the images was NIS Elements.

### Experiment 2: Root tip timelapse

A second experiment was performed to monitor potential diurnal patterns in root O_2_ distribution. After root establishment, devices were transferred to an automated microscope stage. Diurnal patterns in daylight were simulated by installing the same grow light from root establishment above the microscope. Wheat seedlings were *c*. 60 cm from the light source, and a smart timer allowed for integrated light period and dark period imaging cycles. The ‘light period’ was simulated by the light on for 55 min and off for 5 min for 12 consecutive hours. The 5 min off periods during the light period allowed for automated imaging without the grow light interfering with luminescence signals. Additionally, black‐out curtains and foil channeled light to the growing plant and blocked plant roots and the planar optode from direct light exposure. The ‘dark period’ was characterized by 12 h of continuous darkness. Once plants were set to grow on the microscope stage, they were not moved until the end of the experiment.

For each treatment, we imaged a single device containing one to two roots using the same optical configuration as in Experiment 1 (O_2_‐gradient characterization). Individual root tips were imaged every hour (10× magnification, tiling individual pictures) for 19–57 h to monitor root growth rate and hourly variation in O_2_ concentration.

### Image processing

Images were stored as TIFF files. Images were then processed using a combination of imagej and R v.4.2.2 (R Core Team, 2022). Luminescence images were converted to ‘Text Images’ in imagej. Text Images were then converted into O_2_ maps in R, following the described calibration (see Device design, construction, and filling section). Finally, calibrated Text Images were re‐imported to imagej to allow for further analysis.

**Fig. 2:**
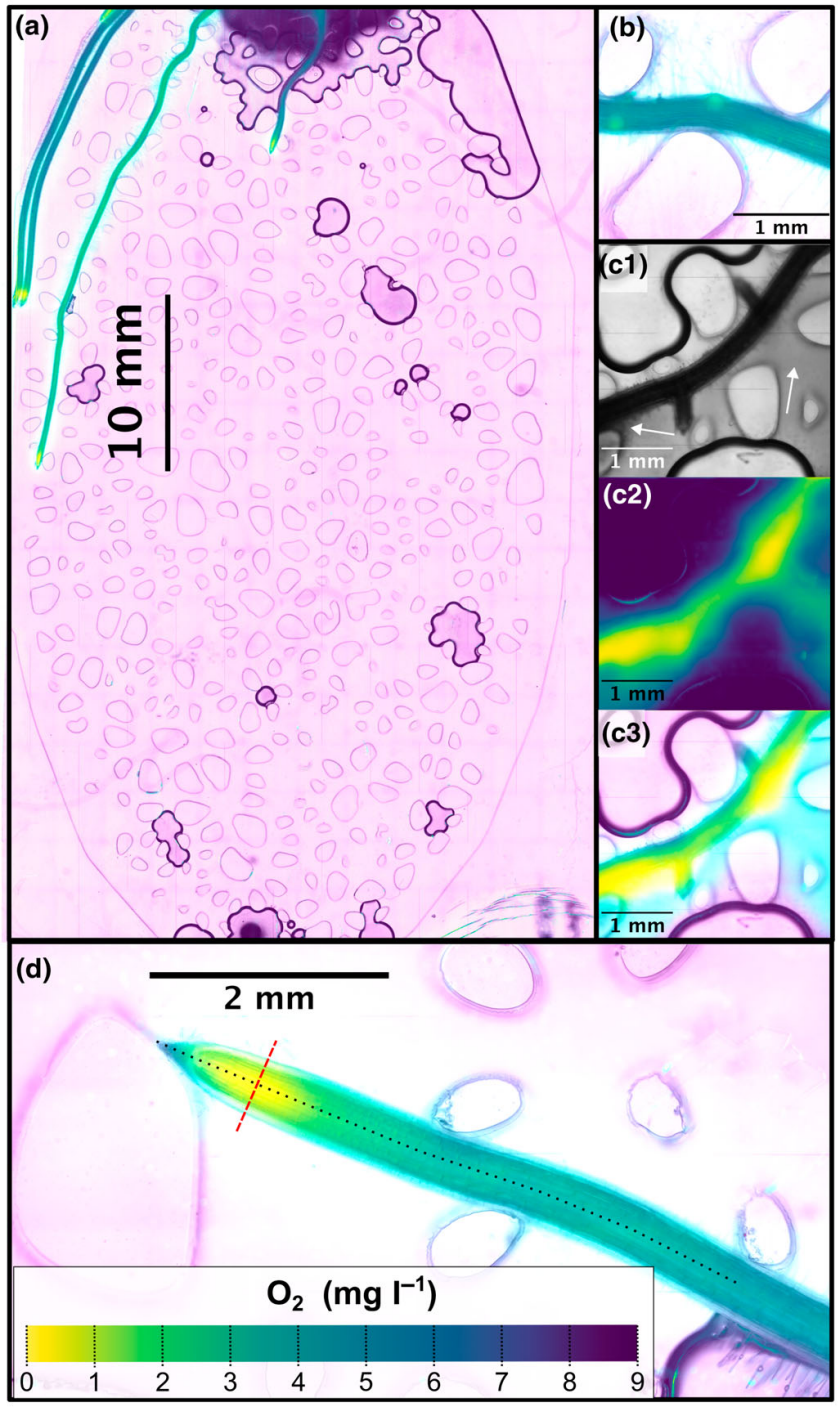
Composite images of phase contrast and O_2_ map of *Triticum aestivum* roots. (a) Soil Community treatment, composite image, 4× magnification; full‐device view of several roots; the dark area at the top of the image is a shadow from the pipette tip. (b) Soil Community, composite, 10×; lateral root emergence. (c) Soil Community, 10×; accumulation of organic matter along the lower edge of the root and subsequent oxygen consumption in zones of lateral root emergence; c1 represents the phase contrast image with darker portions of the image (highlighted with white arrows) denoting areas of organic matter accumulation, c2 the false color O_2_ map, and c3 the composite image. (d) Sterile, composite, 10×; close‐up of a primary root tip; the red dashed line represents an example of a transverse profile and the black dotted line, a longitudinal profile.

Only images of completely submerged roots with no immediate root neighbors were selected for longitudinal and transverse profile analysis (Fig. 2d). All profiles were drawn on phase contrast images using the Profile tool in imagej. Profiles were overlain onto corresponding O_2_ maps to extract O_2_ values along the profiles. For both experiments, we analyzed longitudinal profiles along the root tip, denoting points of interest at the edge of the root cap, the meristem, and the emergence of the first root hairs. For the O_2_‐gradient characterization (Experiment 1), we also defined transverse profiles that spanned from the root growth media, through the visually identified O_2_ minimum, and back into the root growth media; the ends of each transverse profile were defined visually as regions seemingly unaffected by root O_2_ dynamics (i.e. consistent with background O_2_ concentrations).

For O_2_‐gradient characterization (Experiment 1), we calculated the length of transverse and longitudinal profiles that fell below hypoxic (1.92 mg l^−1^) and suboxic (0.16 mg l^−1^) thresholds. For longitudinal profiles, to account for differences in absolute length between the root cap and the first emergence of root hairs, we normalized each profile by dividing the pixel position by the length of the entire cap‐to‐hair region. As a result, normalized profiles represent a general continuum from root cap (normalized position = 0) to the emergence of the first root hairs (normalized position = 1.0). We then compared O_2_ concentrations near more mature root tip sections (normalized positions 0.75–1.0) by averaging the O_2_ concentrations across the range within each profile. Finally, for transverse profiles, we calculated the maximum average decrease between the profile boundary and minimum by subtracting the minimum O_2_ value observed in each profile from the average of the two O_2_ values at the boundary of each profile.

For the root tip timelapse (Experiment 2), longitudinal profiles were analyzed to explore the relationship between growth rate and O_2_ minimum. We calculated the distance between the root cap position in consecutive, 1‐h time‐resolved images. This distance was divided by the time elapsed (i.e. 1 h) to determine the growth rate during the period in which no images were collected. We estimated the instantaneous growth rate of a root in a single image as the mean of the growth rates before and after the image was taken. We then averaged the lowest 500 μm of O_2_ values along the longitudinal profile to derive an average O_2_ minimum to which we could compare growth rates. We chose to use the average of several values instead of a single value to mitigate the potential effects of anomalous pixel values in the profile.

### Statistics

All statistical analyses were performed in R v.4.2.2 (R Core Team, 2022). For Experiment 1 (O_2_‐gradient characterization), we compared longitudinal and transverse profiles across treatments with mixed effects models. We used linear mixed effects models for normally distributed (or appropriately transformed) response variables and generalized linear mixed effects models for non‐normal responses. Mixed effects modeling allowed us to account for inter‐device variance within each treatment and repeated measurements of the same root over time. For each model, the treatment (i.e. Sterile, *P. protegens*, or Soil Community) served as the fixed effect, and each microfluidic device and root identity nested within its device served as random effects. Using these specifications, we analyzed several response variables: for the longitudinal profiles, the average O_2_ concentration from relative positions 0.75–1.00, the length of the hypoxic region, and the length of the suboxic region, and for the transverse profiles, the length of the hypoxic and suboxic regions as well as the maximum change in O_2_ concentration. Results of all mixed effects models for Experiment 1, including the response distribution and link functions, for Experiment 1 are reported in Supporting Information Table S1.

For the timelapse experiment (Experiment 2), we performed a simple linear regression between O_2_ minima and root growth rate and visualized these data. To assess the significance of these relationships, we constructed two linear mixed effects models. In Models 1 and 2, the average O_2_ minimum was the response variable. For Model 1, root growth rate was the fixed effect. For Model 2, root growth rate, treatment, light vs dark period, and the interaction between treatment and light vs dark period served as the fixed effects. In both models, we included a random intercept for each root nested within its treatment. This structure accounts for repeated measurements taken on the same root and reflects the fact that every treatment was applied on a single microfluidic device. Because each treatment was only applied to a single microfluidic device in Experiment 2, the observations obtained from the many roots on that device are technically pseudoreplicates. As a result, the mixed effects models cannot separate a true treatment effect from ordinary device‐to‐device variation. Furthermore, to keep models as simple as possible, we deliberately employed a random intercept formulation, rather than a full repeated measures analysis, to address same‐root measurements over time. Thus, the results of our linear fixed effects models – particularly the estimates for treatment, light–dark periods, and their interactions – should be considered exploratory.

All linear mixed effects models were constructed using the ‘lmer’ function from the lme4 package (Bates *et al*., 2015), with *P*‐values for each random and fixed effect estimated via the Satherwaite approximation in the lmertest package (Kuznetsova *et al*., 2017). All generalized linear mixed models were constructed using the ‘glmmtmb’ function from the glmmtmb package (McGillycuddy *et al*., 2025). All model residuals were inspected for normality either via the Shapiro–Wilk test (base R, linear mixed effects models) or with the ‘simulateResiduals’ function from the DHARMa package (Hartig *et al*., 2024, generalized linear mixed effects models). Finally, conditional and marginal *R* ^2^ values were approximated for Models 1 and 2 using the ‘r.sqauredGLMM’ function from the mumin package (Bartoń, 2025).

## Results

### Emergent patterns of low‐oxygen zones

Across all treatments, we consistently observed low‐O_2_ regions near growing primary root tips (Fig. 2a,d), lateral root tips, and lateral root primordia (Fig. 2b). In a few instances in the Soil Community treatment, we observed low‐O_2_ regions near more mature sections of the root that seemed to coincide with accumulations of particulate organic matter that were co‐extracted with the inoculum (Fig. 2c).

### Longitudinal profiles of root tips

**Fig. 3:**
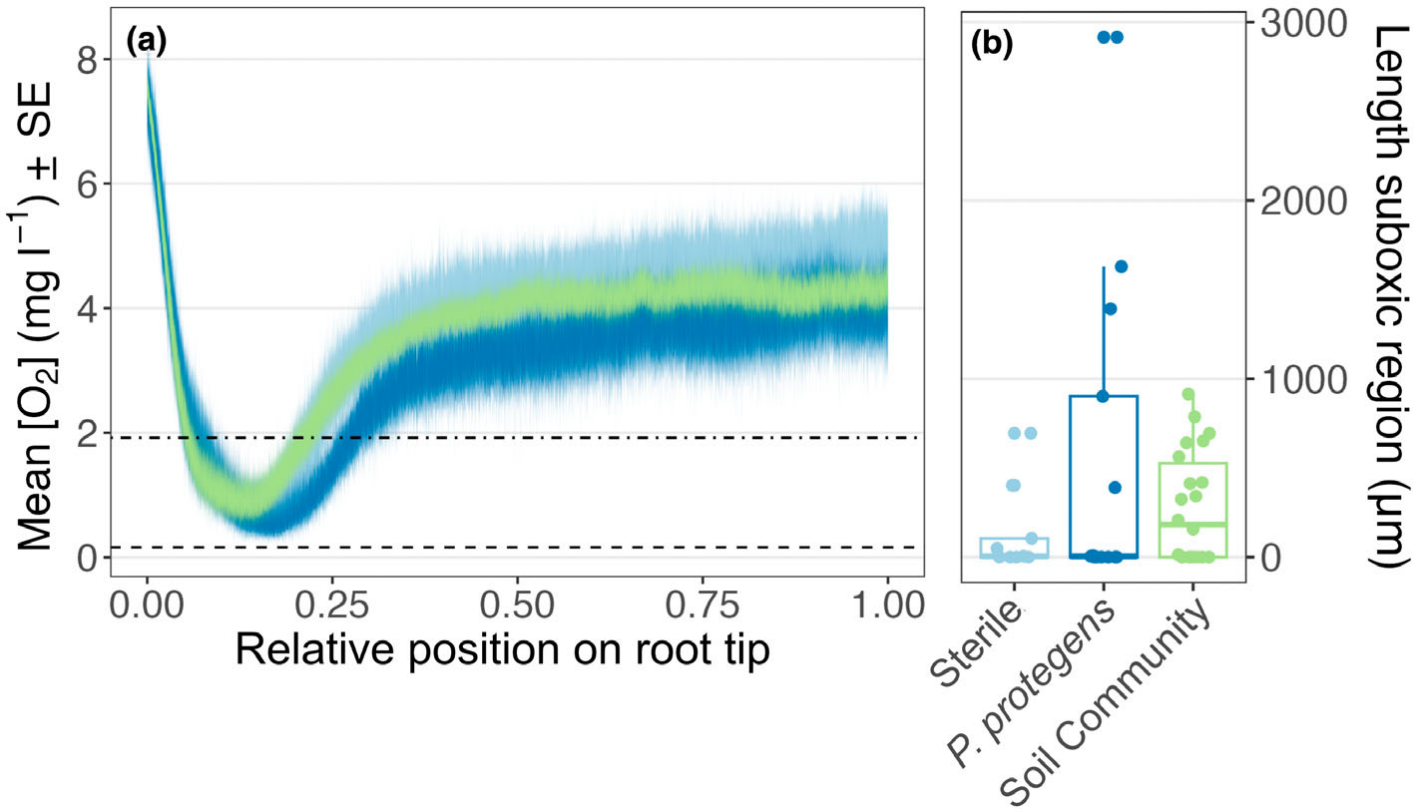
Analysis of *Triticum aestivum* root tip longitudinal profiles. (a) Average longitudinal profiles of O_2_ concentration from root cap (relative position = 0.0) to the first emergence of root hairs (relative position = 1.0) by treatment. Light blue, Sterile; dark blue, *Pseudomonas protegens*; green, Soil Community. Lines represent the mean O_2_ concentration for a given treatment plus or minus the SE. Horizontal dotted lines represent hypoxic (1.92 mg l^−1^) and suboxic (0.16 mg l^−1^) thresholds. (b) Boxplot showing length of suboxic region for longitudinal profiles; individual points represent individual observations; the box shows the interquartile range (IQR); the horizontal line inside the box marks the median, and the whiskers extend to the most extreme observations that lie no farther than 1.5 × IQR below the first quartile and above the third quartile.

Across all treatments, root longitudinal profiles tended to follow a similar pattern (Figs 3a, S1, S2). From the root cap to the emergence of root hairs, O_2_ concentrations began near saturation, declined to some minimum (often located behind the root apical meristem, in the zone of elongation) and then rose to a new constant value, well‐below the value at the root cap (Fig. 3a). This pattern was evident in unscaled, individual observations (Fig. S1) but became remarkably similar when profiles were normalized to length between root cap and root hairs (Figs 3a, S2).

The average O_2_ concentration from relative positions 0.75–1.00 was 4.4 ± 0.2 mg l^−1^ with no significant differences between treatments (Table S1). The average length of the root tip below the hypoxic threshold (in the longitudinal direction) was 1285 ± 211 μm across all three treatments, with no significant difference between treatments (Table S1). Several individual profiles contained regions that dipped below the suboxic threshold (Figs 3b, S1). Across all treatments, the average length of the suboxic region was 332 ± 85 μm. Within individual treatments, the average length of the suboxic region was 139 ± 82 μm for Sterile, 557 ± 253 μm for *P. protegens*, and 278 ± 66 μm for Soil Community. However, there were no significant differences between treatments (Fig. 3b; Table S1).

### Transverse profiles of root tips

**Fig. 4:**
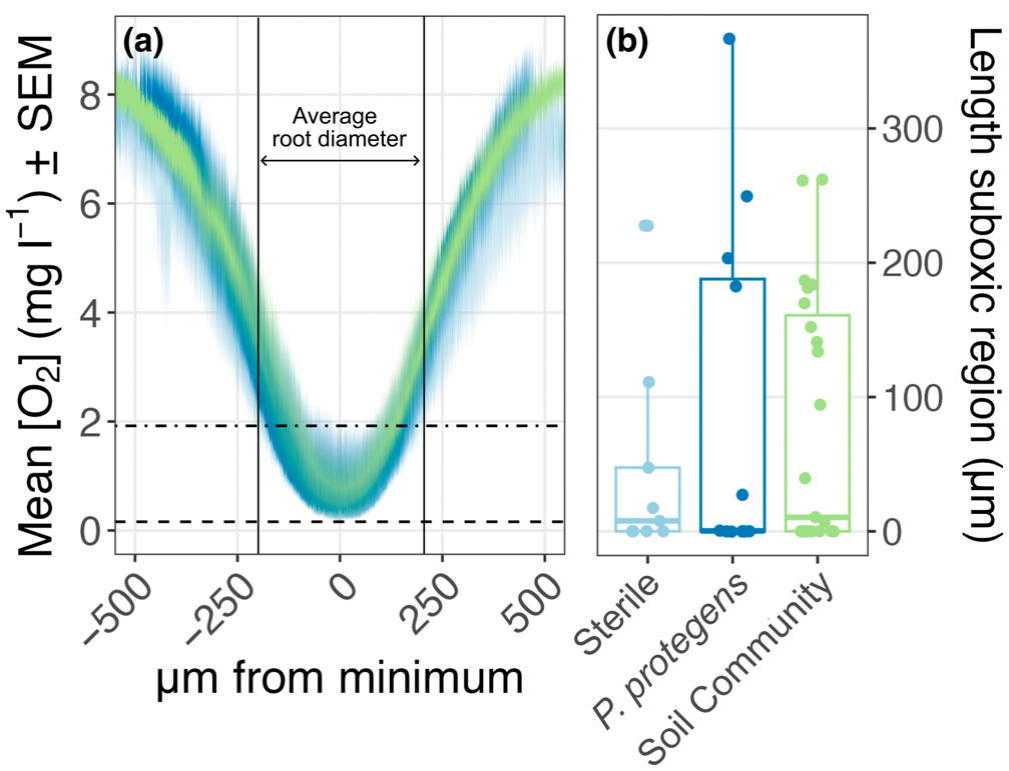
Analysis of *Triticum aestivum* root tip transverse profiles. (a) Average transverse profiles of O_2_ concentration by treatment. Light blue, Sterile; dark blue, *Pseudomonas protegens*; green, Soil Community. Colored lines represent the mean O_2_ concentration for a given treatment plus or minus the SE. Note that all profiles were centered on the profile minimum. Vertical solid lines represent the average root edge position. Horizontal dotted lines represent hypoxic (1.92 mg l^−1^) and suboxic (0.16 mg l^−1^) thresholds. (b) Boxplot showing length of suboxic region for transverse profiles; individual points represent individual observations; the box shows the interquartile range (IQR); the horizontal line inside the box marks the median, and the whiskers extend to the most extreme observations that lie no farther than 1.5 × IQR below the first quartile and above the third quartile.

Transverse profiles exhibited a similar pattern across treatments: in the growth medium, O_2_ concentrations were near saturation, but within 500 μm, we consistently observed local minima below hypoxic and suboxic thresholds (Figs 4a, S3). The average decrease between the transverse profile maximum (at the boundary) and the profile minimum (at the center) was 8.1 ± 0.1 mg l^−1^. There were no differences in the magnitude of this decrease between treatments (Table S1; Fig. S4). The length of hypoxic and suboxic regions in transverse profiles was shorter than that of longitudinal profiles. On average, 290 ± 23 μm of each profile extended below the hypoxic region, and 74 ± 15 μm extended below the suboxic region, with no significant differences between treatments (Fig. 4b; Table S1).

### Timelapse observations of root growth and oxygen consumption

**Fig. 5:**
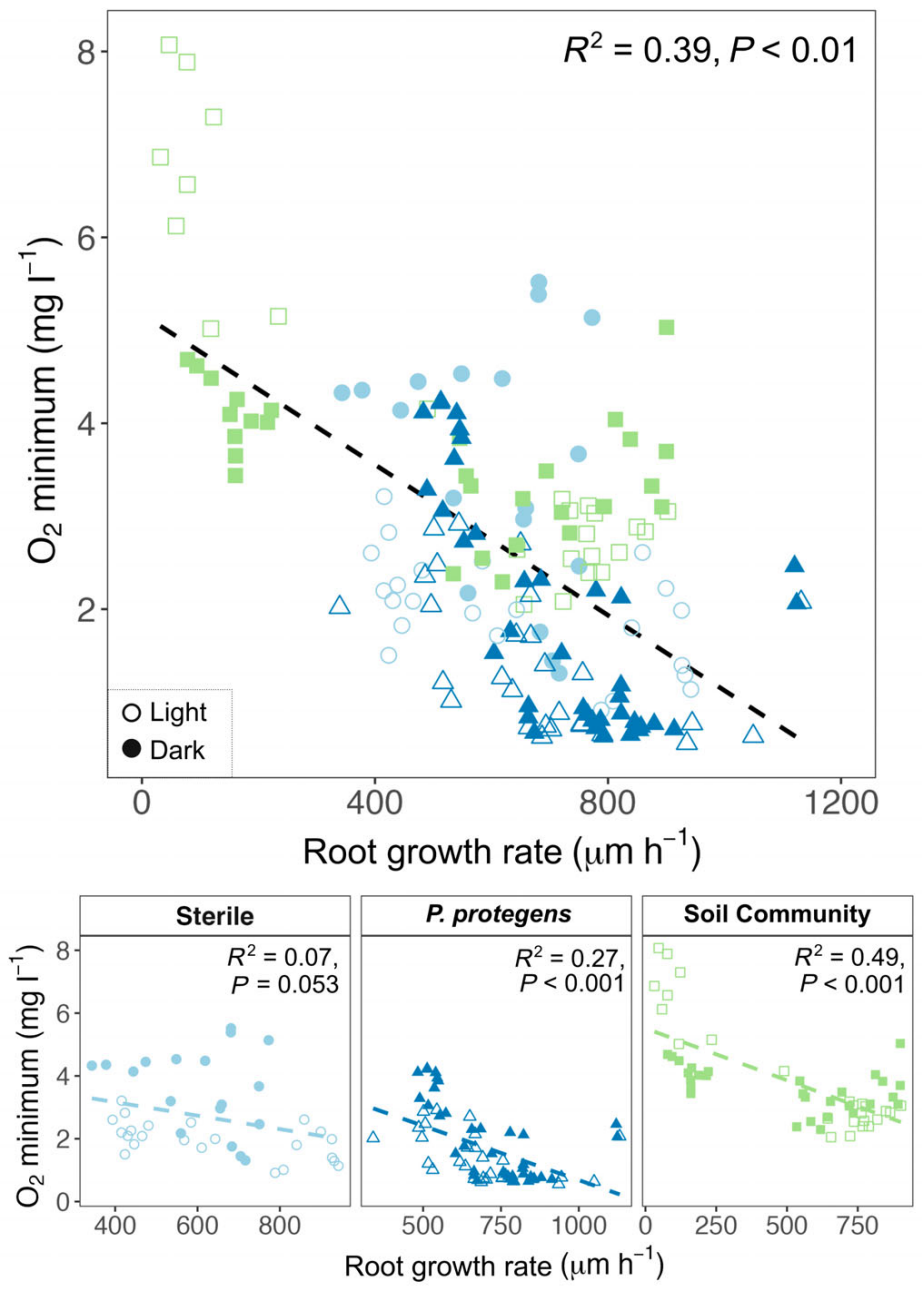
Relationship between *Triticum aestivum* root growth rate and root tip O_2_ minimum. Linear regression results for root growth rate and the average O_2_ minimum observed in timelapse longitudinal profiles. The upper panel represents the entire dataset, whereas the lower panels represent the data separated by treatment: Sterile, *Pseudomonas protegens*, and Soil Community. The dashed line represents the best‐fit line within each panel. Open shapes represent measurements taken during the light period, and filled shapes represent measurements taken during the dark period.

There were no clear diurnal patterns in average O_2_ minima at the root tip (Fig. S5). The average root growth rate in the timelapse experiment was 621 μm h^−1^, and simple linear regression revealed a relationship between root growth rate and root tip O_2_ minimum (Fig. 5) across the entire data set as well as within each treatment. Linear mixed effects modeling revealed that root growth rate could explain 21% of the variance in O_2_ minima, with explanatory power increased to 54% after incorporating random effects (Table 1, Model 1). The fixed effects of Model 2 explained 49% of the variance in O_2_ minima, with explanatory power increasing to 69% after incorporating random effects. In Model 2, root growth rate, light vs dark periods, and the interaction between treatment and light vs dark periods were all significant predictors of O_2_ minima (Table 1, Model 2).

**Table 1:** Results of linear mixed effects models.

Model	Variable	Estimate (SE)	Significance	*R* ^2^_m_ (*R* ^2^_c_)
1	Intercept	4.22 (0.441)	***	0.21 (0.54)
	Growth rate	−2.75 × 10^−3^ (3.52 × 10^−4^)	***	
2	Intercept	3.58 (0.597)	**	0.49 (0.69)
	Growth rate	−2.74 × 10^−3^ (3.19 × 10^−4^)	***	
	Treatment: *Pseudomonas protegens*	−6.42 × 10^−3^ (0.776)		
	Treatment: Soil Community	1.92 (0.926)		
	Light vs Dark : Dark	1.46 (0.281)	***	
	*P. protegens* × Dark	−0.884 (0.355)	*	
	Soil Community × Dark	−1.99 (0.368)	***	

Variables represent solely the fixed effects. Levels of statistical significance are represented by asterisks: *, *P* < 0.05; **, *P* < 0.01; ***, *P* < 0.01. *R* ^2^_c_ = conditional *R* ^2^, that is, the variance explained by both fixed and random effects; *R* ^2^_m_ = marginal *R* ^2^, that is, the variance explained by solely fixed effects.

## Discussion

### Anoxic microsites establish in the rhizosphere

Our results demonstrate that low‐O_2_ conditions reliably establish in the rhizosphere, particularly at the root tip. Consistent with other studies which have shown that roots can decrease O_2_ concentrations at broader scales (Rudolph *et al*., 2012; Bereswill *et al*., 2023), we observed low O_2_ concentrations along the entire plant root relative to the surrounding media. Furthermore, we regularly observed hypoxic and suboxic conditions in the environment around the root tip (Fig. 2a). Likely, the low‐O_2_ conditions at the root tip are an emergent feature of plant root physiology. The highest cellular O_2_ consumption rates (and thus lowest O_2_ concentrations) occur at the root tip, within the quiescent center (Zimmermann *et al*., 2022) or in the zones of cell division and elongation (Mancuso & Boselli, 2002; McLamore *et al*., 2010; Mugnai *et al*., 2012). In fact, the O_2_ demands of root stem cells within the quiescent center are so great that the interiors of these cells are often hypoxic, even under well‐oxygenated conditions (Mira *et al*., 2023). Although others have observed hypoxic conditions *within* root tip cells, our work is the first to characterize micrometer‐scale O_2_ concentrations *exterior to* the root and to directly observe anoxic microsites in the environment around the root tip.

The regular presence of suboxic regions around the root tip suggests that microorganisms in the vicinity of the root tip could perform anaerobic metabolisms, such as denitrification. Assuming the plant root grows tangentially to the O_2_ sensing optode and that root consumption of O_2_ is radial and uniform, our 2D mapping allows us to predict the volume of the suboxic region around each root tip (see Fig. S6). Using the average measured root tip diameter and the average longitudinal and transverse suboxic lengths, we estimate that the growing root tip is surrounded by a *c*. 1.4 × 10^6^ μm^3^ suboxic volume. Soil microaggregates can range from 20 to 50 μm in diameter (Totsche *et al*., 2018), and most soil bacteria range in size from 0.4 to 5 μm (Luan *et al*., 2020). Thus, suboxic microsites at root tips may influence tens of thousands to millions of bacterial cells across multiple microenvironments. Whether these suboxic microenvironments, which co‐occur with the primary zone of nutrient uptake, can sustain anaerobic microbial metabolisms remains a critical but unresolved question for plant nutrient acquisition (see the Implications for our understanding of rhizosphere biogeochemistry section).

Our results also suggest that anoxic microsites likely establish near mature root sections in natural soils. Although we infrequently observed low‐O_2_ zones outside of the root tip, in one case in the Soil Community treatment, organic matter accumulated alongside a more mature root section, resulting in a localized zone of O_2_ depletion (Fig. 2c). This phenomenon was never observed in the Sterile or *P. protegens* treatments, suggesting that microbial communities and soil‐derived organic matter are required for anoxic microsite formation at more mature sections of the root. The low‐O_2_ zone in Fig. 2(c) highlights the potential importance of organic matter diffusion in establishing anoxic microsites. Unlike most of our observations, the root in Fig. 2(c) was positioned near an encroaching bubble. Typically, the devices were almost completely saturated, allowing for maximal diffusion of organic matter and movement of microorganisms away from the root. However, in the presence of bubbles, such as in Fig. 2(c), organic matter diffusion and microbial habitat space are constrained, presumably concentrating O_2_ demand near the root (Yang & van Elsas, 2018; Zech *et al*., 2022) and prompting the formation of an anoxic microsite. Compared to our microfluidic devices, real, unsaturated soils would have thinner and more discontinuous films of water. Thus, anoxic microsites prompted by lack of organic matter diffusion and subsequent localized O_2_ demand – such as that observed in Fig. 2(c) – may be even more prevalent in natural soils. Future experiments should explore how diffusional constraints on microbes and organic matter contribute to anoxic microsite formation at more mature sections of the root.

### Root oxygen consumption drives suboxic conditions at root tip

In the O_2_‐gradient characterization experiment (Experiment 1), the root consumption of O_2_ overshadowed the microbial consumption of O_2_. There were no significant differences between treatments in O_2_ concentration along the root tip or at more mature sections of the root, suggesting that, compared to roots, microbes had minimal influence on the observed O_2_ patterns. Our data are consistent with the findings of Højberg & Sorensen (1993), who reported that root O_2_ demand was 10–30 times greater than microbial O_2_ demand in the rhizosphere (Højberg & Sorensen, 1993). However, counter to our findings, Højberg & Sorensen (1993) were still able to discern microbial O_2_ demand to some extent. The lack of microbial influence in Experiment 1 may be due to a variety of factors.

First, we suspect that the zone of maximal microbial O_2_ consumption did not coincide with the zone of maximal root O_2_ consumption. Microbial rhizosphere colonization tends to peak several mm behind the root cap in ‘older’ sections of the root (Herron *et al*., 2013; Garcia Arredondo *et al*., 2024), whereas we observed maximal O_2_ uptake just behind the apical meristem in young, actively growing portions of the root tip (Figs 2, 3). Possibly, there were no differences in O_2_ concentration across treatments at the root tip because root cell O_2_ consumption overwhelmingly dominates O_2_ consumption in these microenvironments where there are relatively few microbes present. However, even in our examination of more mature sections of the root, such as the analysis of relative positions 0.75 to 1 in the longitudinal profiles (*c*. 5–7.5 mm behind the root tip), we saw no difference in O_2_ concentrations, suggesting other and/or additional factors must have concealed microbial O_2_ consumption.

Second, due to microscope slide size constraints, we only observed young and potentially poorly colonized wheat roots. Future studies of larger wheat plants with more mature roots and rhizosphere communities may reveal more pronounced microbial effects on rhizosphere O_2_ concentrations.

Third, the near‐total hydration of the devices could have rendered root‐derived organic matter and microorganisms relatively diffuse (see the Anoxic microsites establish in the rhizosphere section), obfuscating the influence of microorganisms. In the Soil Community treatment, the organic matter co‐extracted with the microbial community would have allowed proliferation of microorganisms relatively far from the root. In a more realistic experimental system with forced spatial constraints (i.e., smaller pore sizes and unsaturated conditions), a greater contribution of microbial respiration to O_2_ depletion may have been observed.

Finally, our device design allowed for relatively rapid O_2_ diffusion. PDMS is an O_2_‐permeable polymer (Li *et al*., 2024), and the thickness of our devices (*c*. 500 μm) would have allowed for rapid diffusion of O_2_ through the PDMS walls and growth medium. We used uncoated PDMS to ensure the media was bulk oxic (a prerequisite to anoxic microsite formation), to minimize root hypoxic stress, and to conservatively identify O_2_ consumption hotspots. Though in similar experimental systems, bacterially driven anoxic microsites were detectable only when the PDMS was coated with an O_2_‐impermeable glue (Ceriotti *et al*., 2022). Furthermore, the microfluidic device did not allow for compaction of soil particles as one would expect in a natural soil (Mueller *et al*., 2024). Thus, the rates of O_2_ diffusion inside the microfluidic devices were likely much greater than rates expected in natural soil, and our devices likely underrepresent anoxic microsite formation in the rhizosphere.

### Root growth rate, light vs dark period, and the presence of microbes collectively determine root tip oxygen minima

One of the most evident drivers of O_2_ minima at the root tip was growth rate, with greater growth rates resulting in lower O_2_ values (Fig. 5; Table 1). Linear mixed effects modeling allowed us to explore how growth rate, light vs dark periods, and treatment collectively influenced the O_2_ minima at the root tip (Table 1). However, future work with replicated devices per treatment and an explicit temporal correlation structure will be needed to confirm these findings. Treatment alone had no significant effect on O_2_ minima, underscoring the relatively small influence of microbes on O_2_ concentrations in our system. Across all treatments, dark period O_2_ values tended to be greater than light period O_2_ values. From a microbial perspective, this result is unsurprising as previous studies have found rhizosphere microorganisms to be more active and deplete local O_2_ concentrations during light periods (i.e., the day; Herron *et al*., 2013; Garcia Arredondo *et al*., 2023). However, the light vs dark period effect also existed for the Sterile treatment, suggesting that the effect is at least partially driven by patterns in root O_2_ consumption. There is no clear consensus on how root respiration rates vary throughout the day. Older studies of root respiration found greater respiration rates, and thus O_2_ consumption, during periods of photosynthesis (Huck *et al*., 1962; Hansen, 1980). However, more recent works find a highly variable lag between peak photosynthesis and peak root respiration (Edwards & McLaughlin, 1978; Kuzyakov & Gavrichkova, 2010; Savage *et al*., 2013; Song *et al*., 2015; Lai *et al*., 2016). In our system, root O_2_ consumption was generally higher (i.e., lower O_2_ concentrations) during light periods (i.e., periods of photosynthesis), though quantifying the exact lag between light periods and root respiration was beyond the scope of this study.

Interestingly, within the interaction terms of Model 2, we observe our first evidence of microbial influence on O_2_ concentrations in this study. After accounting for differences in growth rate and diurnal patterns in root O_2_ consumption, the interaction between light vs dark periods and treatment had a significant effect on root tip O_2_ concentration (Table 1). Specifically, compared to the Sterile treatment, the presence of *P. protegens* and soil community inocula inhibited nightly increases in root tip O_2_ concentration (Table 1). We hypothesize that the microbial consumption of residual root exudates led to greater overall O_2_ consumption during dark periods within inoculated treatments. Thus, microbe‐containing treatments exhibited less of a difference in O_2_ minima between light and dark periods. The combined effects of root growth rate, light vs dark period, and treatment may partially explain why we were unable to discern differences between treatments in Experiment 1, which captured solely daily images. Thus, these measurements did not account for differences in instantaneous growth rate and only captured images during photosynthetic periods. Future studies may need to consider all these dynamics in order to fully characterize the root vs microbial controls on rhizosphere O_2_ concentrations.

### Implications for our understanding of rhizosphere biogeochemistry

To our knowledge, this study is the first to use microfluidics and integrated optode sensors to provide high‐resolution 2D spatial maps of O_2_ concentrations in the rhizosphere. These maps clearly demonstrate that anoxic microsites can consistently establish in the rhizosphere, particularly near plant root tips. Our findings provoke questions as to whether these anoxic microsites can prompt anaerobic microbial metabolisms in the vicinity of the plant root. If root‐tip‐associated anoxic microsites host denitrification processes, local inorganic nitrate concentrations could decrease, thus, limiting plant nitrogen availability (Lacroix *et al*., 2023). Additionally, if root‐tip‐associated anoxic microsites host reductive dissolution of Fe‐oxides, the phosphorus, metals, and carbon bound to those Fe‐oxides could become available to plants and microorganisms (Liptzin & Silver, 2009; Huang & Hall, 2017; Malakar *et al*., 2020). For example, arsenic solubilized during Fe‐oxide dissolution in anoxic microsites can bioaccumulate in upland crops, such as corn (Malakar *et al*., 2020). Additionally, carbon liberated from Fe‐oxide dissolution in the rhizosphere can result in enhanced microbial access to carbon and, subsequently, higher carbon dioxide emissions from rhizosphere soil (Bölscher *et al*., 2025; Lacroix *et al*., 2025).

Our results also suggest that the contributions of root vs microbial O_2_ demand must be further defined, particularly in more soil‐like systems. Microbial and root respiration will likely respond differently to regional changes in climate. For instance, root respiration rates, and thus root O_2_ consumption, are generally expected to increase with warming but may have varied degrees of response (Atkin *et al*., 2000; Jian *et al*., 2022; Ryhti *et al*., 2022). Root exudation rates and subsequent microbial O_2_ consumption may increase or decrease with warmer temperatures and drought conditions (Leuschner *et al*., 2022; Jiang *et al*., 2023; Brunn *et al*., 2025), depending on the biome. Although we did not measure root exudation in this study, others have successfully employed exudate measurements in similar experimental systems (Aufrecht *et al*., 2022; Novak *et al*., 2024). Pairing root exudate measurements with planar O_2_ sensors is a promising next step in studying root‐associated anoxic microsite dynamics. Above all, improving predictions of root and microbial O_2_ demand in the rhizosphere will improve our ability to predict changes in root‐associated anoxic microsites and their downstream biogeochemical impacts.

Finally, the relationship between root O_2_ consumption and growth rate prompts questions about whether the suboxic region observed at the root tip is stationary for long enough to provoke anaerobic metabolisms. If a root is growing relatively quickly, it is more likely to have a lower O_2_ minimum, but that O_2_ minimum is less likely to be in the same place for an extended period. For example, the average suboxic region length in the once‐daily images was 332 μm, and the average growth rate in the timelapse experiment was 621 μm h^−1^. Under these conditions, a stationary soil microorganism would experience *c*. 32 min of suboxic conditions as the root tip grows past. In some experimental systems, denitrifiers require multiple hours of anoxia to meaningfully upregulate nitrate and nitrous oxide reductases at the community level (Lycus *et al*., 2018). However, by some accounts, *c*. 30 min may be sufficient time to activate nitrate and nitrous oxide reductases in denitrifying bacteria (Baumann *et al*., 1996) if these reductases are not already active (Marchant *et al*., 2017). Regardless, our findings suggest that changes in root growth speed could change the resulting average O_2_ minimum and, potentially, metabolic pathways around the root tip.

The examination of rhizosphere anoxic microsites is nascent, yet our study suggests that root and microbial respiration dictate the formation and dynamics of anoxic microsites in the rhizosphere. Quantifying the biogeochemical impact of these root‐associated anoxic microsites on the fate of rhizosphere carbon and plant nutrient and contaminant acquisition should remain an urgent topic of research.

## Competing interests

None declared.

## Author contributions

EML: writing – review and editing, writing – original draft, visualization, project administration, methodology, funding acquisition, investigation, formal analysis, data curation, conceptualization. GC: writing – review and editing, methodology, funding acquisition, conceptualization. DG‐S: writing – review and editing, methodology, conceptualization. SMB: writing – review and editing, methodology. JSB: writing – review and editing. CK: writing – review and editing. PdA: writing – review and editing, methodology. MK: writing – review and editing, conceptualization, funding acquisition, supervision.

## Disclaimer

The New Phytologist Foundation remains neutral with regard to jurisdictional claims in maps and in any institutional affiliations.

## Supporting information


**Fig. S1** Unaveraged root tip longitudinal profiles by treatment and raw distance.
**Fig. S2** Unaveraged root tip longitudinal profiles by treatment and normalized distance.
**Fig. S3** Unaveraged root tip transverse profiles by treatment.
**Fig. S4** Decrease in O_2_ concentration between transverse profile boundaries and transverse profile minima.
**Fig. S5** Average O_2_ minimum at root tip by time of day.
**Fig. S6** Diagram showing presumed radial extent of suboxic conditions from plant roots.
**Table S1** Table showing results of linear mixed effects models for Experiment 1.
Please note: Wiley is not responsible for the content or functionality of any Supporting Information supplied by the authors. Any queries (other than missing material) should be directed to the *New Phytologist* Central Office.

## Data Availability

Code and data from this study are available on GitHub at https://github.com/elacroix92/microfluidics.
