# Supplementary material for: Root and microbial contributions to anoxic microsite formation in the rhizosphere: a microfluidic approach
Source: New Phytol. 2026 Mar 20;250(5):3486–98. doi: 10.1111/nph.71109 (PMC13150310; doi:10.1111/nph.71109)
Supplement: Supplementary file 1 — Fig. S1 Unaveraged root tip longitudinal profiles by treatment and raw distance. Fig. S2 Unaveraged root tip longitudinal profiles by treatment and normalized distance. Fig. S3 Unaveraged root tip transverse profiles by treatment. Fig. S4 Decrease in O2 concentration between transverse profile boundaries and transverse profile minima. Fig. S5 Average O2 minimum at root tip by time of day. Fig. S6 Diagram showing presumed radial extent of suboxic conditions from plant roots. Table S1 Table showing results of linear mixed effects models for Experiment 1. Please note: Wiley is not responsible for the content or functionality of any Supporting Information supplied by the authors. Any queries (other than missing material) should be directed to the New Phytologist Central Office. [file NPH-250-3486-s001.pdf]

New Phytologist Supporting Information

Article title:

Root and microbial contributions to anoxic microsite formation in the rhizosphere: a microfluidic approach

Authors:

Emily M. Lacroix[1,2], Giulia Ceriotti[1], Daniel Garrido Sanz[3,4], Sergey M. Borisov[5], Jasmine S. Berg[1], Christoph Keel[3], Pietro de Anna[6], Marco Keiluweit[1]

Affiliations:

[1] University of Lausanne, Institute of Earth Surface Dynamics, 1015 Lausanne, Switzerland

[2] Department of Earth Science, Dartmouth College, Hanover, NH, 03755, USA

[3] University of Lausanne, Department of Fundamental Microbiology, 1015 Lausanne, Switzerland

[4] Universidad Autónoma de Madrid, Department of Biology, 28049 Madrid, Spain

[5] Graz University of Technology, Institute of Analytical Chemistry and Food Chemistry, 8010 Graz, Austria

[6] University of Lausanne, Institute of Earth Sciences, 1015 Lausanne, Switzerland

*Co-corresponding authors: Emily Lacroix (emily.lacroix@dartmouth.edu) and Marco Keiluweit (marco.keiluweit@unil.ch)

Article Acceptance Date: February 28, 2026

Supplementary Table S1. ***Results of linear mixed effects models for Experiment 1, gradient characterization experiment.*** Variables represent solely the fixed effects. Column p represents the p-values estimated for each fixed effect. Significant p-values are in bold. Link functions are specified solely for generalized linear mixed effects models.

**Profile**	**Response Variable**	**Response Distribution**	**Link function**	**Fixed-Effects**	**p**
Longitudinal	Average O_2, relative positions 0.75-1.0	Gaussian	NA	Intercept	**<0.01**
				Treatment: *P. protegens*	0.39
				Treatment: Soil Community	0.56
	Length hypoxic region	Tweedie	log	Intercept	**<0.01**
				Treatment *P. protegens*	0.41
				Treatment: Soil Community	0.80
	Length suboxic region	Tweedie	Log	Intercept	**<0.01**
				Treatment: *P. protegens*	0.94
				Treatment: Soil Community	0.50
Transverse	Length hypoxic region	Gaussian	NA	Intercept	**<0.01**
				Treatment: *P. protegens*	0.85
				Treatment: Soil Community	0.64
	Length suboxic region	Tweedie	log	Intercept	**<0.01**
				Treatment: *P. protegens*	0.73
				Treatment: Soil Community	0.50
	Max. average decrease	Gaussian	NA	Intercept	**<0.01**
				Treatment: *P. protegens*	0.41
				Treatment: Soil Community	0.42

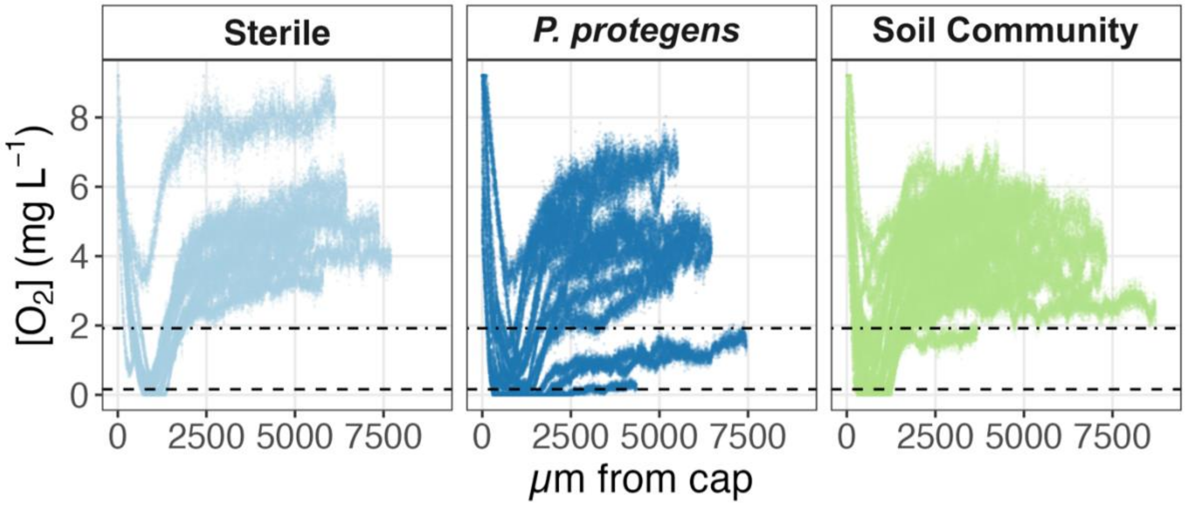

Supplementary Figure S1. ***Unaveraged root tip longitudinal profiles by treatment and raw distance.*** Each pixel within a unique replicate is represented by a single circle. Horizontal lines represent oxygen regimes defined in Berg et al. (2022). Hypoxic = 1.92 mg L^{-1} ; suboxic = 0.16 mg L^{-1}.

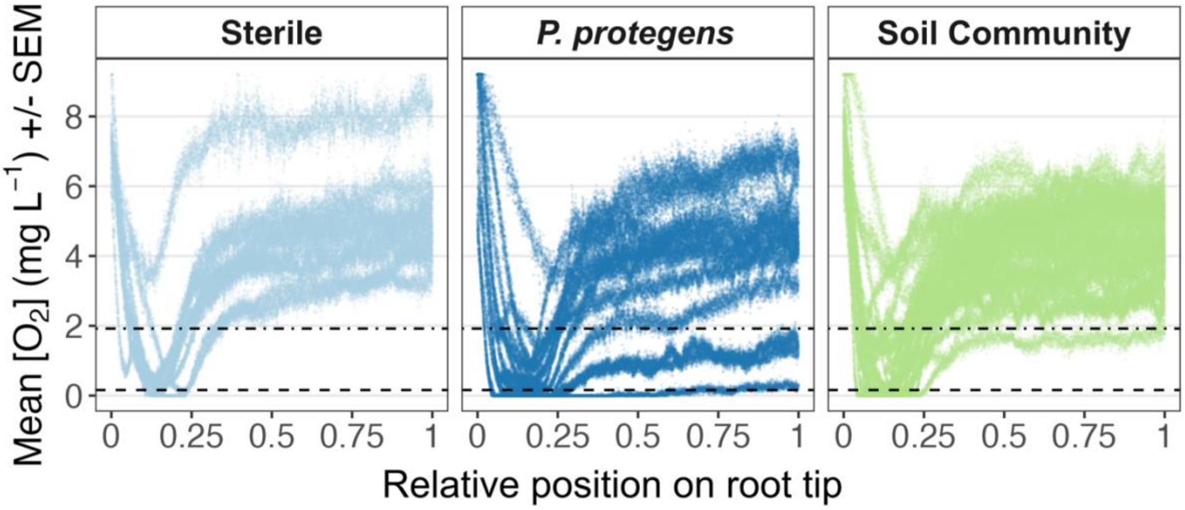

Supplementary Figure S2. ***Unaveraged root tip longitudinal profiles by treatment and normalized distance.*** Each pixel within a unique replicate is represented by a single circle. Horizontal lines represent oxygen regimes defined in Berg et al. (2022). Hypoxic = 1.92 mg L^{-1} ; suboxic = 0.16 mg L^{-1}.

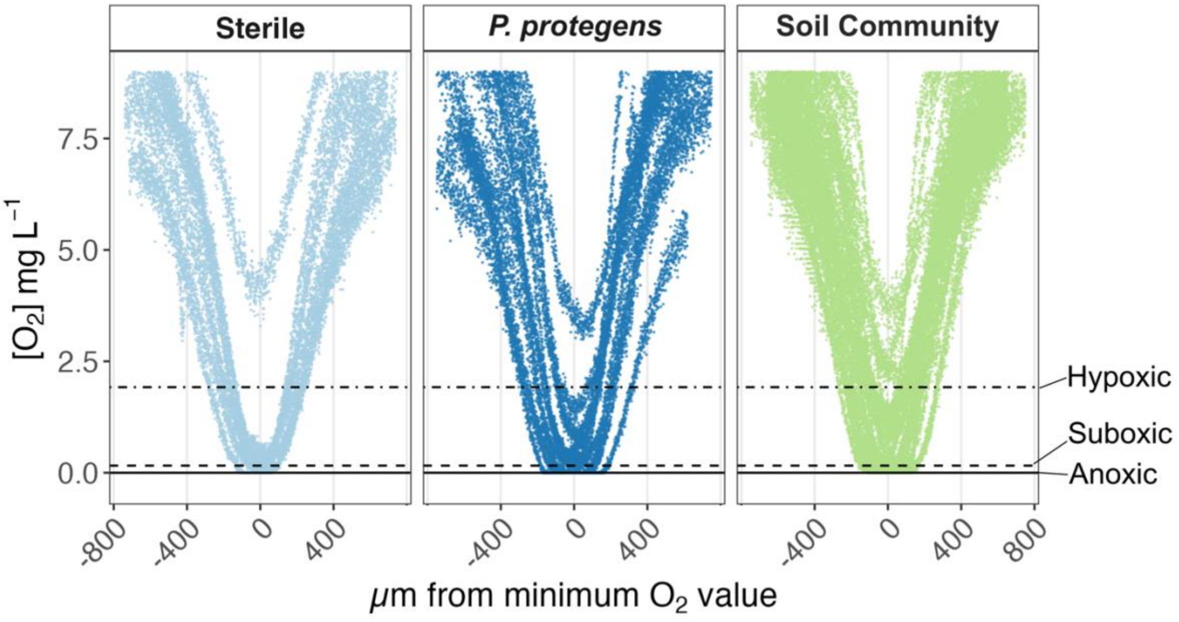

Supplementary Figure S3. ***Unaveraged root tip transverse profiles by treatment.*** Each pixel within a unique replicate is represented by a single circle. Each profile is centered to the profile minimum. Horizontal lines represent oxygen regimes defined in Berg et al. (2022). Hypoxic = 1.92 mg L^{-1} ; suboxic = 0.16 mg L^{-1}, and anoxic = 9.6 x 10^{-6} mg L^{-1}.

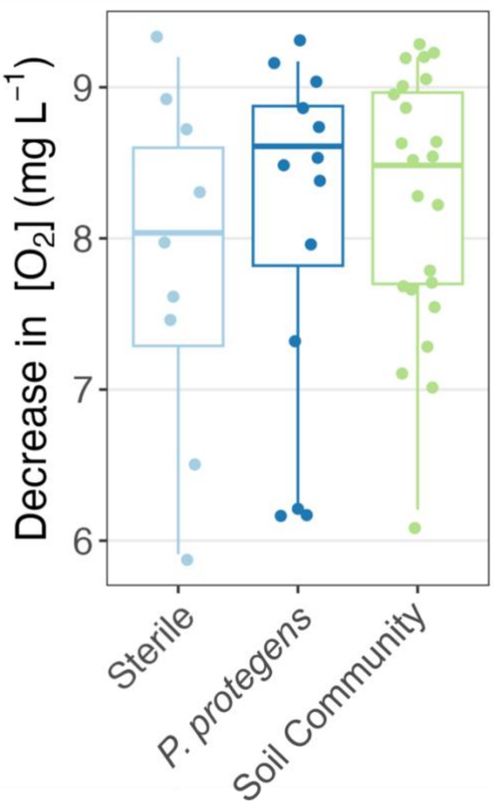

Supplementary Figure S4. ***Decrease in O_2 concentration between transverse profile boundaries and transverse profile minima.*** Data are represented as boxplots with individual points representing individual observations. The box shows the interquartile range (IQR); the horizontal line inside the box marks the median, and the whiskers extend to 1.5 x IQR below the first quartile and above the third quartile.

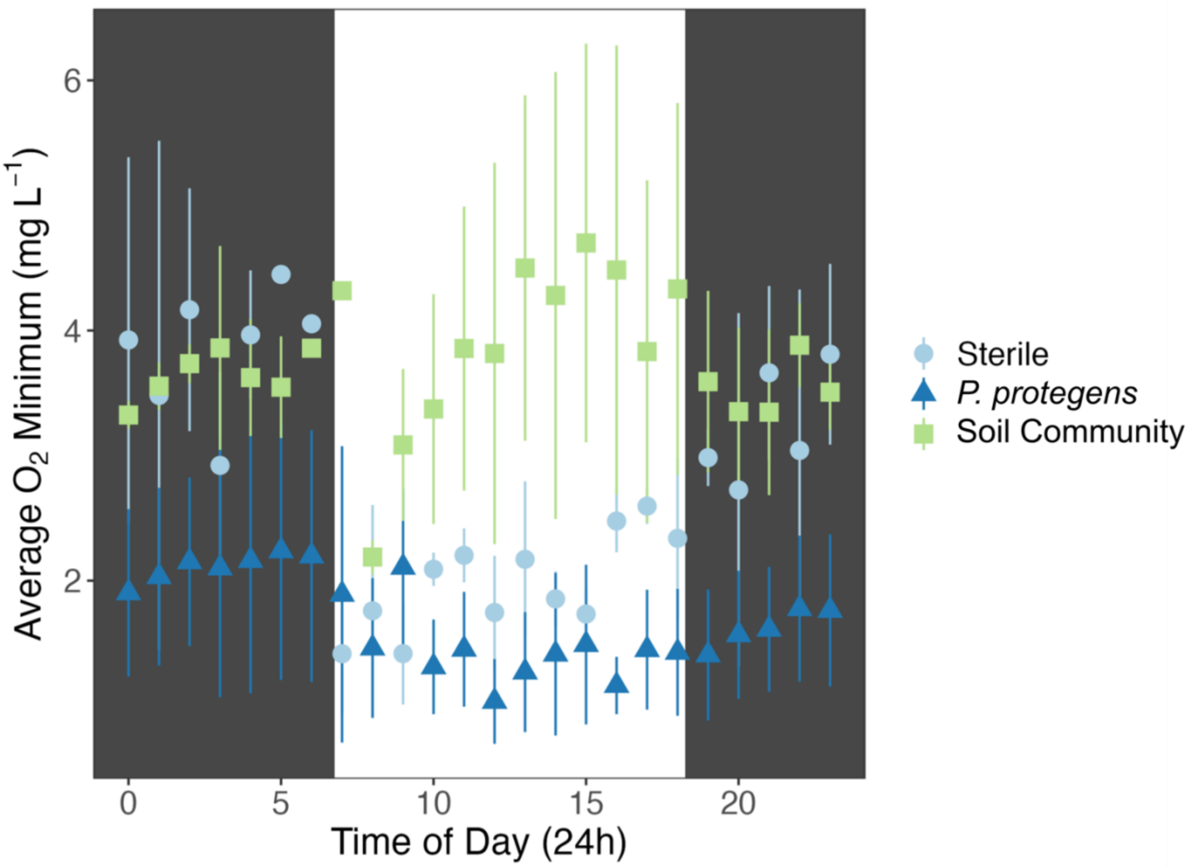

Supplementary Figure S5. ***Average O_2 minimum at root tip by time of day.*** The white panel represents the hours during which the grow light was on (i.e., light period), and the dark panels represent the hours during which the grow light was off (i.e., dark period). Symbols represent the mean of replicate observations, and the error bars represent the standard error of the mean.

Hypothetical Suboxic Volume

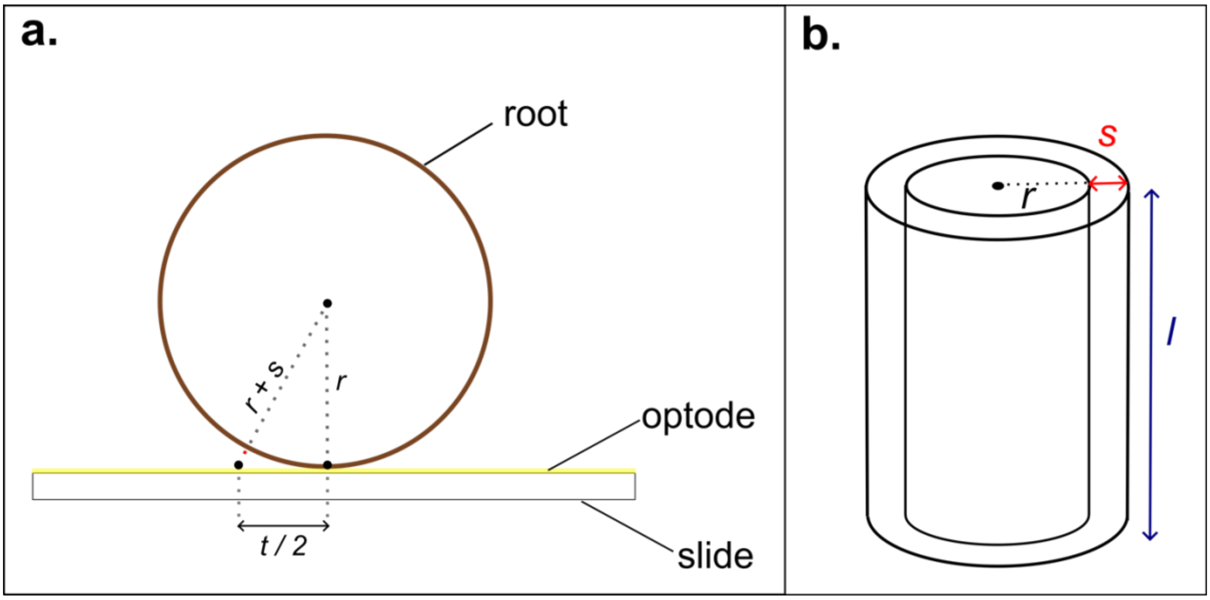

Supplementary Figure S6. ***Diagram showing presumed radial extent of suboxic conditions from plant roots***. Where r = root radius, s = radial extent of suboxic region, t = length of suboxic region in transverse profile. and l = the length of suboxic region in the longitudinal profile.

Calculating s

$$\left({}^{t}/_{2}\right)^2 + r^2 = (r+s)^2$$

$$r + s = \sqrt{\left({}^{t}/_{2}\right)^2 + r^2}$$

$$s = \sqrt{\left({}^{t}/_{2}\right)^2 + r^2} - r$$

$$s = \sqrt{\left({}^{t}/_{2}\right)^2 + r^2} - r$$

$$s = \sqrt{\left({}^{74\,\mu m}/_{2}\right)^2 + 200\,\mu m^{\,2}} - 200\,\mu m = 3.4\,\mu m$$

Root Volume

$$V_{root} = \pi \times r^2 \times l = \pi \times 200\,\mu m^{\,2} \times 332\,\mu m = 4.172 \times 10^7\,\mu m^3$$

Suboxic Rhizosphere Volume

$$V_{rhizo} = (\pi \times (r+s)^2 \times l) - V_{root} =$$

$$(\pi \times (200\,\mu m + 3.4\,\mu m)^2 \times 332\,\mu m) - 4.172 \times 10^7\,\mu m^3 = 1.43 \times 10^6\,\mu m^3$$

References

Berg, J.S., Ahmerkamp, S., Pjevac, P., Hausmann, B., Milucka, J., Kuypers, M.M.M., 2022. How low can they go? Aerobic respiration by microorganisms under apparent anoxia. FEMS Microbiology Reviews 46, fuac006. doi:10.1093/femsre/fuac006
